# The evaluation of short fusion in idiopathic scoliosis

**DOI:** 10.4103/0019-5413.58603

**Published:** 2010

**Authors:** Wiwat Wajanavisit, Patarawan Woratanarat, Thira Woratanarat, Kitti Aroonjaruthum, Noratep Kulachote, Wajana Leelapatana, Wichien Laohacharoensombat

**Affiliations:** Department of Orthopedics, Faculty of Medicine Ramathibodi Hospital, Mahidol University, Thailand; 1 Department of Preventive and Social Medicine, Faculty of Medicine, Chulalongkorn University, Thailand; 2 Department of Family Medicine, Faculty of Medicine Ramathibodi Hospital, Mahidol University, Thailand

**Keywords:** Idiopathic scoliosis, short fusion, selective posterior fusion, pedicle screw and plate fixation

## Abstract

**Background:**

Selective thoracic fusion in type II curve has been recommended by King *et al.* since 1983. They suggested that care must be taken to use the vertebra that is neutral and stable so that the lower level of fusion is centered over the sacrum. Since then there has been the trend to do shorter and selective fusion of the major curve. This study was conducted to find out whether short posterior pedicle instrumentation alone could provide efficient correction and maintain trunk balance comparing to the anterior instrumentation.

**Materials and Methods:**

A prospective study was conducted during 2005-2007 on 39 consecutive cases with idiopathic scoliosis cases King 2 and 3 (Lenke 1A, 1B), 5C and miscellaneous. Only the major curve was instrumented unless both curves were equally rigid and of the same magnitude. The level of fusion was planned as the end vertebra (EVB) to EVB fusion, although minor adjustment was modified by the surgeons intraoperatively. The most common fusion levels in major thoracic curves were T6–T12, whereas the most common fusion levels in the thoraco-lumbar curves were T10–L3. Fusion was performed from the posterior only approach and the implants utilized were uniformly plate and pedicle screw system. All the patients were followed at least 2 years till skeletal maturity. The correction of the curve were assessed according to type of curve (lenke IA, IB and 5), severity of curve (less than 450, 450-890 and more than 900), age at surgery (14 or less and 15 or more) and number of the segment involved in instrumentation (fusion level less than curve, fusion level as of the curve and fusion more than the curve)

**Results:**

The average long-term curve correction for the thoracic was 40.4% in Lenke 1A, 52.2% in Lenke 1B and 56.3% in Lenke 5. The factors associated with poorer outcome were younger age at surgery (<11 years or Risser 0), fusion at wrong levels (shorter than the measured end vertebra) and rigid curve identified by bending study. However, all patients had significant improved trunk balance and coronal hump at the final assessment at maturity. Two patients underwent late extension fusion because of junctional scoliosis.

**Conclusions:**

With modern instrumentations, the EVB of the major curve can be used at the end of the instrumentation in most cases of idiopathic scoliosis. In those cases with either severe trunk shift, younger than 11 years old, or extreme rigid curve, an extension of one or more levels might be safer. In particular situations, the concept of centering the lowest vertebra over the sacrum should be adopted.

## INTRODUCTION

Selective thoracic fusion in type II curve has been recommended by King *et al*,^1^ since 1983. They suggested that care must be taken to use the vertebra that is neutral and stable so that the lower level of fusion is centered over the sacrum. Yukihiro M *et al*,^2^ in 2006 defined the predicted stable vertebra (PSV) that was centered over the sacrum on traction roentgenogram. Luk *et al*,^3^ selected the lower level by their technique of fulcrum bending (FB). These authors concluded they could save one or more motion segments comparing to the previous reports. Since 2001, the Lenke system of classification has been popularized as an accurate mean to determine which vertebrae should be fused.^4^ This classification system requires analysis of the upright coronal and sagittal radiographs along with the supine side bending radiographic views. The triad classification system consists of a curve type (1–6), a lumbar spine modifier (A, B, C), and a sagittal thoracic modifier (-, N, +). All three regions of the radiographic coronal and sagittal planes, the proximal thoracic, main thoracic and thoracolumbar/lumbar are designated as either the major curve (largest Cobb measurement) or minor curves, which are categorized into structural and nonstructural types. The recommendations are that the major and structural minor curves are included in the instrumentation and fusion, and the nonstructural minor curves are excluded.

However, in 2004, Lenke and associates reported satisfactory results for selective thoracic fusion in 44 cases with main thoracic and the minor lumbar C modifier curves.^5^ Many authors advocated selective short anterior fusion in thoracolumbar and thoracic curves by various methods.^6^–^10^ These general trends to limit the fusion segments in idiopathic scoliosis are the basis and rationale for this study in order to find out whether short posterior pedicle instrumentation alone could provide efficient correction and maintain trunk balance.

## MATERIALS AND METHODS

A prospective study of idiopathic scoliosis cases with a King 2 and 3 (Lenke 1A, 1B) and the thoracolumbar curve in Lenke 5 during 2005–2007 was conducted. The inclusion criteria comprised idiopathic juvenile and adolescent scoliosis, 9–20 years of age, and complete follow-up data with standing X-rays at 2 years or more post-operation. The exclusion criteria were all other types of scoliosis, patients declining to undergo selective short instrumentation at the thoracic (T) and thoracolumbar (TL) levels utilizing the pedicle screws and plates (Ramathibodi spinal system Manufactured by: T.K.S. Metal Works LTD, 2 soi Bangwag,16;Bangwag, Pasicharoen, Bangkok 10160, Thailand).

Fusion was performed from the posterior only approach and the implants utilized were uniformly plate and pedicle screw system. Only the major curve was instrumented unless both curves were equally rigid and of the same magnitude. The level of fusion was planned as the end vertebra (EVB) to EVB fusion, although minor adjustment was modified by the surgeons intraoperatively. All the patients were followed at least for 2 years till skeletal maturity.

Since 1990, the de-rotation technique of correction of scoliosis has been routinely adopted in Ramathibodi Hospital by pedicle plates and screws. In 2005, a series of 25 cases was analyzed to evaluate the efficacy of apical de-rotation.^11^ In these cases, the selection of levels of fusion was based on the balanced vertebrae at the upper and caudal end of the curve. These cases were regarded as the control for this study.

Preoperative and the last follow-up Cobb's angle measurement was recorded by two chief residents, the average figure was recorded. Patients with a plump line deviation of more than 1.5 cm were recorded as imbalanced posture. Any complications or revisions were also recorded. The correction of the curve was assessed as type of curve (Lenke IA, IB and 5), severity of curve (less than 45°, 45°-89° and more than 90°), age at surgery (14 years or less and 15 years or more) and number of the segment involved in instrumentation (fusion level less than curve, fusion level as of the curve and fusion more than the curve) Student t-test was used to evaluate the efficacy of curve correction between pairs of variables. One-way analysis of variance was performed for more than two-group comparison of continuous data. All statistical analyses were done by using STATA 10.0 (STATA Corp, Texas).

## RESULTS

There were 43 cases that underwent apical de-rotation during the period of study. Thirty-nine cases met the criteria of short selective instrumentation of the major curve. Analysis of the preoperative and the last postoperative Cobb's angle were shown in Table 1. The patient's mean age at the time of surgery was 14.7 years (range 9 to 20 years). The mean follow-up is 2.1 years (range 0.8 to 3 years). The distributions of the curve as classified by Lenke were as follows: 1A (n = 12), 1B (n = 10), 5C (n = 10) and miscellaneous (n = 7). The average pre-operative Cobb's angle was 52.8° and the average final post-operative Cobb's angle was 25.4°. The residual Cobb's angle is 48.11% when compared with pre-operatively. This degree of correction was inferior to our previous study^12^ by using the balanced vertebrae as the end of instrumentation (48 versus 59%).

**Table 1:** Baseline characteristics of cases shows the curve type, preoperative and postoperative Cobb's angle, level of fusion and % correction achieved

Case#	Age (in years)	Major curve	Lenke type	Pre-op Cobb (in degrees)	Post-op Cobb (in degrees)	Fusion level	Remark (correction, %)
1	16	T4–L1	1A	33	20	T5–T12	39.3
2	10	T7–L1	1A	38	29	T7–L1	23.6
3	20	T6–T12	1A	40	15	T7–T12	62.5
4	15	T3–T12	1A	35	15	T3–T12	57.1
5	17	T5–T12	1A	36	24	T5–T12	33.3
6	14	T4–T12	1A	38	29	T4–T12	23.6
7	13	T5–T12	1A	35	20	T5–L1	42.8
8	9	T5–L1	1A	65	35	T5–T12	46.1
9	11	T5–L1	1A	67	50	T5–T11	25.3
10	13	T4–T12	1A	52	24	T4–T11	53.6
11	15	T4–T12	1A	60	35	T4–T12	41.7
12	12	T4–T12	1A	90	58	T5–T12	35.5
Mean	13.75			49.08€	29.5€		40.43
SD	3.11			17.91	13.33		12.96
13	14	T4–T11	1B	33	13	T5–T11	60.6
14	15	T5–T12	1B	38	22	T6–T12	42.1
15	19	T3–T11	1B	38	5	T3–T10	86.8
16	14	T5–T12	1B	34	24	T6–T12	29.4
17	12	T6–T12	1B	30	25	T6–T12	16.6
18	18	T6–T12	1B	67	25	T5–T12	62.7
19	15	T4–T12	1B	45	15	T4–T12	66.6
20	18	T4–T12	1B	45	26	T4–T12	42.2
21	15	T4–L1	1B	60	14	T5–T12	76.6
22	15	T5–T12	1B	97	60	T5–T12	Revision
Mean	15.5			48.7€	22.9€		52.20
SD	2.17			20.72	14.74		22.03
23	18	T9–L2	5C	40	22	T10–L2	45.0
24	20	T10–L2	5C	45	20	T10–L2	55.5
25	14	T9–L4	5C	50	15	T10–L2	70.0
26	14	T9–L4	5C	40	10	T11–L4	75.0
27	14	T10–L3	5C	40	15	T10–L3	62.5
28	13	T9–L4	5C	40	20	T10–L3	50.0
29	12	T9–L4	5C	41	28	T10–L3	31.7
30	16	T6–L3	5C	45	15	T5–L2	66.6
31	16	T11–L4	5C	50	20	T11–L3	60.0
32	16	T10–L3	5C	47	24	T12–L3	48.9
Mean	15.3			43.8€	18.9€		56.53
SD	2.41			4.15	5.23		13.01
*P*-value*	0.2424			0.7103	0.1275		0.0709
33	13	T3–T11	2A	56	33	T3–T10	41.0
34	11	T6–T12	2C	90	50	T6–T12	44.4
35	12	T4–L1	3B	65	24	T4–L1	63.1
36	17	T11–L3	3C	46	23	T11–L3	50.0
37	13	T3–T11	3C	96	65	T5–T11	32.3
38	14	T5–T12	3C	100	48	T5–T12	52.0
39	14	T10–L3	6C	92	55	T10–L3	40.2
Mean	13.4	7.71		77.9	42.6	7.14	46.1
SD	0.72			8.2	6.1		3.8

€ Significant difference between pre-op and post-op with *P*-value <0.05 by using paired t-test

* *P*-value comparing among Lenke IA, IB, and 5 groups by using one-way analysis of variance

### Factors affecting the surgical outcome

#### 1) Curve magnitude

The patients with curve larger than 90° had significantly poorer results when compared with those of smaller curve. The mean value of correction in the coronal plane for group I (n = 17) (<45°) was 48%, group II (n = 16) (45°–89°) was 54%, and group III (n = 6) (>90°) was 39% respectively. The *P*-value between group III and group I was 0.009 and between group III and group II was 0.0015, respectively.

#### 2) Age at surgery

Those patients underwent surgery at the age below 14 years (n = 21) ended up with poorer results than those over 15 years of age (n = 18). The amount of correction in the younger age group was 43.5%, whereas those older age group was 53.0% [Table 2]. The *P*-value between both groups

**Table 2:** Age vs. degrees of correction

Age	Pre-op Cobb (angle in degree)	Post-op Cobb (angle in degree)	% correction (correction of curve in %)
9	65	35	46.15
10	38	29	23.68
11	90	50	44.44
11	67	50	25.37
12	30	25	16.67
12	41	28	31.71
12	90	58	35.55
12	65	28	56.92
13	40	20	50.00
13	35	20	42.85
13	96	65	32.29
13	52	24	53.84
13	56	33	41.07
14	33	13	60.61
14	34	24	29.41
14	40	10	75.00
14	40	15	62.50
14	38	29	23.68
14	92	55	40.21
14	100	48	52.00
14	50	15	70.00
Mean	56.76€	32.10€*	43.45
SD	23.70	16.04	16.02
15	38	22	42.10
15	35	15	57.14
15	90	60	33.33
15	45	15	66.67
15	60	14	76.67
15	60	35	41.67
16	33	20	39.39
16	45	15	66.67
16	50	20	60.00
16	47	24	48.94
17	36	24	33.33
17	46	23	50.00
18	40	22	45.00
18	67	25	62.68
18	45	26	42.22
19	38	5	86.84
20	40	15	62.50
20	45	20	55.55
Mean	47.78€	22.22€*	53.93
SD	13.98	11.39	14.82

€ Significant difference between pre-op and post-op with *P*<0.05

* Significant difference between age group with *P*<0.05

3) The number of vertebral segments involved in the instrumentation unit was shown in Table 3. There were 22 short fusion cases involving 1–2 levels of vertebral segments lesser than the vertebral segments involved in the curve. Fourteen cases involved the same number whilst 7 cases involving 1–2 more vertebral segments. The mean corrections in coronal plane were 48.85, 48.88 and 45.41%, respectively. There was no significant statistical difference.

**Table 3:** Number of vertebrae involved in fusion vs curve correction

Fusion < Curve (n = 22)		Fusion = Curve (n = 14)		Fusion > Curve (n = 7)		*P* value*
		
Cobb (degree)		Cobb (degree)		Cobb (degree)		
			
Pre-op	Post-op	Pre-op	Post-op	Pre-op	Post-op	
33	20	30	25	38	5	
33	13	35	18	38	29	
38	22	35	15	35	20	
34	24	36	24	115	75	
40	22	38	29	96	65	
40	10	65	24	100	48	
40	15	45	20	50	15	
40	20	60	35			
41	28	92	55			
97	60	90	50			
90	60	65	24			
90	58	45	15			
65	35	46	23			
67	50	40	15			
45	15					
46	23					
50	20					
52	24					
56	33					
45	26					
60	14					
50	15					
**Mean Cobb**		**Mean Cobb**		**Mean Cobb**		
		
**Pre-op**	**Post-op**	**Pre-op**	**Post-op**	**Pre-op**	**Post-op**	
52.36	27.59	51.57	26.57	67.43	36.71	
SD		SD		SD		
18.85	15.55	20.06	12.36	34.70	26.48	
*P*€		*P*€		*P*€		
<0.0001		<0.0001		0.0014		
**Mean difference**		**Mean difference**		**Mean difference**		
24.77		19.25		30.00		0.1919	
SD		SD		SD				
8.91		11.37		15.87				
**% correction Mean (SD)**		**% correction Mean (SD)**		**% correction Mean (SD)**			
48.85 (14.98)		48.85 (14.98)		48.92 (22.52)		0.6369	

* P-value compared among fusion groups by using one-way analysis of variance

€ *P*-value compared between pre-op and post-op by using paired *t*-test

#### 4) Lenke's type and curve correction

**Figure 1:**
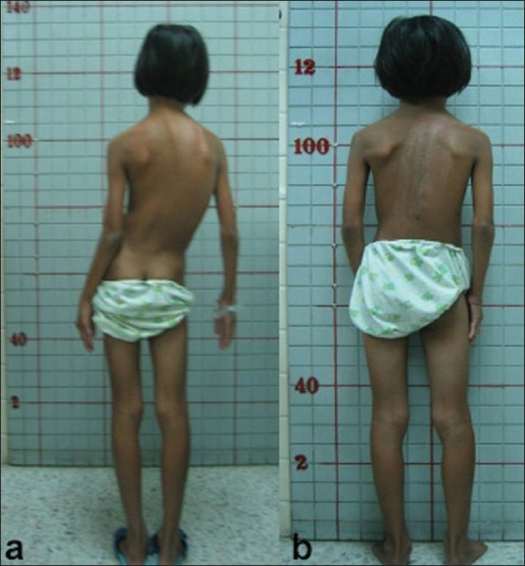
(a) Preoperative clinical picture of case 8, a 9 years old girl with a curve of 60° (b) The postoperative picture revealed a wellbalanced posture. The final curve measures 35°

**Figure 2:**
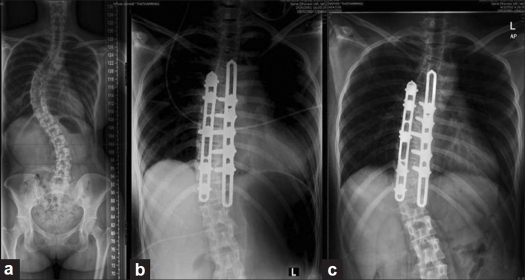
(a) A case of Lenke 1B curve. The preoperative Cobb was 60°. The EVB were T5 and L1 (b) Early postoperative film revealed the instrumentation extending from T6 to T12, one level above the EVB. The curve was corrected to 30° (c) This 2-year follow-up film showed the curve progressing to 35°

**Figure 3:**
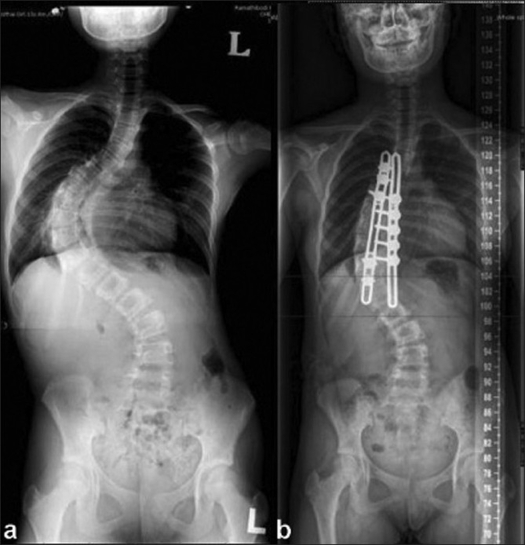
A case of Lenke 1B of 97°. (a) The preoperative film revealed the T curve ranging from T5 to L1. (b) The postoperative film revealed fixation extending from T5 to T12, one level short of the thoracic curve. The postoperative angle was 60°. Extension of fusion was considered

**Figure 4:**
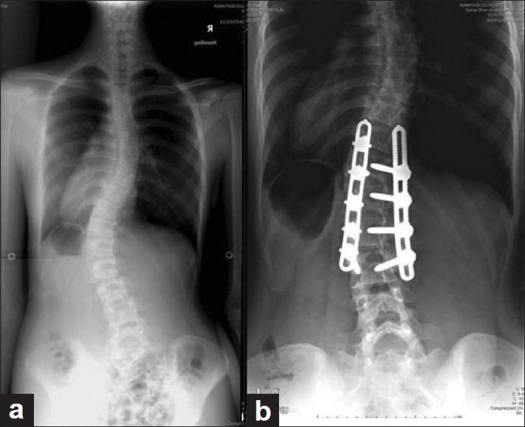
(a) Lenke 5C type of curve. The EVB were T10 and L3 (b) Post-operative film demonstrating T11-l3 fixation. The post-operative angle was 24° against the pre-operative angle of 47°

According to Table 1, the numbers of cases with short fusion, in decreasing order, were 1A (12 cases), 1B (10 cases), 5 (12 cases) and miscellaneous (7 cases). The percentages of curve correction were 40.4, 52.2, 56.5 and 46.1, respectively. There was significant difference between the Lenke 1A group and Lenke 5 group (*P* = 0.008), whereas no significant difference in the Lenke 1A and 1B group was observed (*P* = 0.135). Examples of case from pre-operative to post-operative conditions were shown in Figures 1a–4b.

### Complications

There was one case with late infection requiring implants removal (case #37 of Table 1). The curve was still stable after the implant was removed.

There were two cases where the progressive curve at the lower junction necessitated revision. One belonged to the 1B group (case #22) and the other in 3C group (case #37 of Table 1).

## DISCUSSION

Surgeons performing scoliosis surgery usually encounter two basic questions. Firstly, whether or not all the structural curves should be fused. Secondly, how many vertebral segments should be included in the fusion construct? To address the first question, most surgeons agree that both double major curves should be fused. Through Lenke's meticulous 3D classification,^4^ he advocated to include minor structural curves in the fusion mass. His recommendation is widely adopted, although with modern fixation devices, there is a general trend to do selective fusion over the major curve only. Based on our limited data, it seemed that short fusion over the thoraco-lumbar curve in Lenke 5C type of curve, comparing to Lenke 1A, did well in light of degrees of curve correction and trunk balance [Tables 1 and 2].

In addition, by comparing with our previous series, it seemed that EVB to EVB fusion or less did poorer than utilizing the balanced vertebrae as the end of the fusion mass (curve correction of 48% against 59%). However, we could not demonstrate the difference in degree of curve correction among cases with longer or shorter than EVB to EVB instrumentation in the present series [Table 3]. Instead, we found strong associations of the degree of curve correction with curve magnitude, Lenke's type and the age at surgery, although the latter was not as strong as the formers.

By comparing to anterior instrumentation, Burton *et al*,^12^ found that the posterior pedicle instrumentations were also effective in terms of the degrees of curve correction, balance and number of the vertebrae instrumented. Halm *et al*,^13^ and Monney and Kaelin^14^ had also reported excellent result in short posterior instrumentation for thoraco-lumbar scoliosis. They achieved about 64% correction of the major curve. In our series, we got 56% correction in the Lenke 5C group. Most of our cases had the fusion ended at EVB-1, one level less than the recommendation given by the authors. Yang and Chen^15^ warned for attention of proximal kyphosis if the proximal junction had the kyphotic angle of more than 10 degrees. However, we had not encountered this problem.

Regarding the major thoracic curve, Betz *et al*,^16^ reported that an average of 2.5 lumbar levels can be saved with anterior instrumentation. Suk *et al*,^17^ achieved 61% correction in selective thoracic fusion. They found that the adding-on phenomena occurred in patients who were fused two levels short of the neutral vertebra. This was also our experience and we included our postoperative Cobb measurement to the farthest add-on vertebra. Hamzaoglu *et al*,^18^ used intraoperative halo-femoral traction in those cases of severe thoracic scoliosis (>100 degrees) and could get an average curve correction of 51%. In our series, we got only 39% correction in this particular group. It seems that we need some additional procedures for this kind of problems.

## CONCLUSION

Short fusion can be safely performed in Lenke 5C type of curvature. Short EVB to EVB fusion can be an option in those patients with flexible moderate thoracic curve when the patient's age reaches puberty. Those cases with severe rigid thoracic curve, the concept of neutral vertebra as the base of fusion should be adopted.
